# Bone morphogenetic protein pathway responses and alterations of osteogenesis in metastatic prostate cancers

**DOI:** 10.1002/cnr2.1707

**Published:** 2022-08-19

**Authors:** Meredith D. Provera, Desiree M. Straign, Parvanee Karimpour, Claire L. Ihle, Philip Owens

**Affiliations:** ^1^ Department of Pathology University of Colorado, Anschutz Medical Center Aurora Colorado USA; ^2^ Walsh University Canton Ohio USA; ^3^ Department of Veterans Affairs, Research Service, Eastern Colorado Health Care System Aurora Colorado USA

**Keywords:** blastic, bone, bone morphogenetic protein, lytic, metastasis, prostate cancer

## Abstract

**Background:**

Prostate cancer is a common cancer in men that annually results in more than 33 000 US deaths. Mortality from prostate cancer is largely from metastatic disease, reflecting on the great strides in the last century of treatments in care for the localized disease. Metastatic castrate resistant prostate cancer (mCRPC) will commonly travel to the bone, creating unique bone pathology that requires nuanced treatments in those sites with surgical, radio and chemotherapeutic interventions. The bone morphogenetic protein (BMP) pathway has been historically studied in the capacity to regulate the osteogenic nature of new bone. New mineralized bone generation is a frequent and common observation in mCRPC and referred to as blastic bone lesions. Less common are bone destructive lesions that are termed lytic.

**Methods:**

We queried the cancer genome atlas (TCGA) prostate cancer databases for the expression of the BMP pathway and found that distinct gene expression of the ligands, soluble antagonists, receptors, and intracellular mediators were altered in localized versus metastatic disease. Human prostate cancer cell lines have an innate ability to promote blastic‐ or lytic‐like bone lesions and we hypothesized that inhibiting BMP signaling in these cell lines would result in a distinct change in osteogenesis gene expression with BMP inhibition.

**Results:**

We found unique and common changes by comparing these cell lines response and unique BMP pathway alterations. We treated human PCa cell lines with distinct bone pathologic phenotypes with the BMP inhibitor DMH1 and found distinct osteogenesis responses. We analyzed distinct sites of metastatic PCa in the TCGA and found that BMP signaling was selectively altered in commons sites such as lymph node, bone and liver compared to primary tumors.

**Conclusions:**

Overall we conclude that BMPs in metastatic prostate cancer are important signals and functional mediators of diverse processes that have potential for individualized precision oncology in mCRPC.

## 1 INTRODUCTION

Bone morphogenetic proteins (BMPs) are a class of signaling molecules within the TGF‐β (transforming growth factor‐β) family that signal via downstream mediator SMAD proteins. During signal transduction, BMP ligand dimers bind to type I and type II serine/threonine receptor monomers, resulting in the formation of a hetero‐tetrameric kinase complex. Following ligand‐receptor formation, the constitutively active type II receptors phosphorylate the type I receptors to activate the latter's kinase domains.^1^ Activated type I receptors signal via the SMAD family, a group of downstream mediator proteins that are divided into three functional classes. Regulatory SMADs (R‐SMADs), which include SMAD1, ‐5, and ‐9, are phosphorylated by the activated type I receptors. Phosphorylated R‐SMADs subsequently activate the single common‐mediator SMAD (Co‐SMAD), SMAD4, to form a mature transcription factor. Together, the R‐SMAD‐Co‐SMAD complex translocate to the nucleus to regulate the expression of BMP target genes.^2^ The third class of SMADs, the inhibitory SMADs (I‐SMADs), which include SMAD6 and ‐7, are intracellular BMP pathway antagonists; they act by competing with R‐SMADs 1, ‐5, and ‐9 at the type I receptor binding site and with SMAD4 for complex formation with SMAD1.^3^

BMP signaling is associated with a wide variety of human cancers, including prostate, breast, colorectal, lung, ovarian, and others.^4^ However, the specific effect of BMPs on tumorigenesis and metastasis can be dependent upon the unique BMP ligand and cancer type.^4^, ^5^ For example, in lung cancer, decreased BMP expression from normal tissues and high expression of many BMPs is associated with better overall survival.^6^ However, in human oral squamous cell carcinoma, BMP2 driven epithelization was suppressed by TGFβ, illustrating dynamic control of cell behavior.^7^ BMPs have shown that their ability as morphogens can help epithelial cells expand toward terminal differentiation, and this can be important in reversing early epithelial to mesenchymal transition and accelerating MET for the outgrowth of epithelial cells at distant sites.^8^ In breast cancer, BMP4 signaling to Smad7 was found to suppress metastases by sensitizing cells to anoikis.^9^ BMPs as secreted factors also play diverse roles in many unique tissues in the body as non‐cell autonomous regulators of cell behavior that can be utilized to facilitate cancer growth.^10^ Moreover, BMP ligands have been demonstrated to serve dual antiproliferative and pro‐metastatic roles in cancer both in vivo and in vitro.^11^ Thus, it is evident that BMPs have the dual potential to act as tumor suppressors and tumor promoters, with the specific effects dependent upon the BMP ligand and cancer type.^12^

The BMP pathway has long been studied in prostate cancer due to the role of BMPs in the unique prostate cancer tropism to metastasize in bone. In prostate cancer, BMP signaling has been directly correlated with tumor development and metastasis. For example, BMP6 was detected in over 50% of prostate samples from metastatic adenocarcinoma patients, while expression was not observed in non‐metastatic tumors or benign prostate tissue.^13^ Moreover, BMP6 was found to promote tumor invasion and migration of prostate cancer cells, potentially through the activation of ID‐1 and matrix metalloproteinases (MMPs).^14^ BMP7 expression was detected in metastatic prostate cancer, with increased levels found in castration‐resistant versus androgen dependent prostate cancer, suggesting that the ligand plays a role in the metastatic process. However, BMP7 also demonstrated microenvironment and tumor‐dependent antiproliferative effects both in vivo and in vitro.^15^ On the other hand, BMP2 expression is decreased in prostate cancer compared to benign prostate tissue, and BMP receptor expression is often lost during tumor progression.^5^ However, in vitro, BMP2 was found to enhance C4‐2B prostate cancer cell invasiveness and mediate TNF‐α‐induced invasion.^16^ These studies set the stage for continued understanding of the BMP pathways in metastatic prostate cancer for specific context and therapeutic management.

Metastatic prostate cancers have a unique propensity to colonize bone, where they most often exhibit an osteoblastic phenotype characterized by increased osteoblast activity and bone lesions. The high affinity of prostate cancer for bone is the result of multiple interactions between the tumor and pre‐metastatic site in a non‐cell autonomous manner.^17^ The discovery of aberrant BMP expression in prostate cancer and the recognition of its propensity for bone has led to the hypothesis that BMP signaling promotes bone metastasis via crosstalk between the primary tumor and bone prior to metastasis.^18^ For example, WNT signaling was found to induce BMP4 and BMP6 expression in prostate cancer cells, which in turn stimulated osteoblast differentiation.^19^ In human prostate LNCaP cells, BMP4 induced the production of Sonic hedgehog (SHH), which in turn stimulated BMP receptor and SMAD1 expression in mouse stromal cells to enhance sensitivity to the original BMP4 ligand; together, BMP4 and SHH stimulation also induced the expression of osteoblastic markers.^20^ These findings suggest that BMPs secreted by prostate cancer cells play a role in the induction of osteoblast differentiation and upset the balance of bone remodeling, thus contributing to the osteoblastic phenotype.^18^

In our study, we demonstrate the nuanced expression of BMP signaling using recent online data from cBioPortal and the Cancer Cell Line Encyclopedia (CCLE).^21^, ^22^, ^23^ Additionally, we present the pharmacologic sensitivity to BMP inhibition in a broad array of human prostate cancer cell lines and their known effects on in the bone microenvironment. As mentioned previously, prostate cancer has a propensity to metastasize to bone, where it primarily exhibits a blastic phenotype in the form of blastic or sclerotic bone lesions that stimulate abnormal growth.^24^ A minority of bone metastases display a lytic phenotype, which is characterized by bone destruction, while others appear to be a mix between lytic and blastic phenotypes.^25^ In a previous study, we identified distinct differences in immune cell populations and bio signaling pathways between the lytic and blastic microenvironments of bone metastatic sites.^26^ Of the six prostate cell lines studied, the C42B, MDA PCA 2B, and VCaP lines exhibited blastic‐like phenotypes, while the PC3, 22RV1, and DU145 lines displayed lytic‐like phenotypes. By studying the effects of BMP inhibition in both blastic‐ and lytic‐like models, we seek to elucidate the differential roles of BMP signaling in the two metastatic phenotypes and potentially identify therapeutic targets for metastatic prostate cancer.

## 2 MATERIALS AND METHODS

### 2.1 Cell culture

The PC‐3 (Catalog Number CRL‐1435), 22Rv1 (Catalog Number CRL‐2505), DU145 (Catalog Number HTB‐81), VCaP (Catalog Number CRL‐2876), and C4‐2B (Catalog Number CRL‐3315) cell lines were obtained from the American Type Culture Collection (ATCC) and cultured in Dulbecco's Modified Eagle Medium (DMEM) (Catalog Number 10‐017‐CV) high glucose with sodium pyruvate (Corning), 10% fetal bovine serum (FBS) (Catalog Number 35‐015‐CV) (Corning) and Antibiotic (Catalog Number A5955‐100ML) and Antimycotic Solution (penicillin, streptomycin and amphotericin) (Sigma‐Aldrich). The MDA PCa 2b (Catalog Number) was obtained from the ATCC and cultured in F‐12K Medium (ATCC Catalog Number 30‐2004) with 20% FBS (Corning), Cholera Toxin (Sigma Catalog Number C8052), mouse Epidermal Growth Factor (EGF) (ThermoFisher Catalog Number PMG8041), O‐Phosphoethanolamine (Catalog Number P503), Hydrocortisone (Catalog Number H0888), Sodium Selenite (Catalog Number S5261), human recombinant insulin (Catalog Number 91077C) and Antibiotic and Antimycotic Solution (Sigma‐Aldrich). All cell lines were routinely tested for mycoplasma infection by PCR and authenticated with the short tandem repeat analysis done by the University of Colorado Cancer Center Cell Technologies Shared Resource. Cells were plated at 5.0 × 10^5^ in each well of a six well tissue culture plate. Once cells reached a confluence of ~90% they were treated with 10 μM DMH1 in DMSO (Selleckchem), for 24 h and RNA was collected.

### 2.2 Gene expression

The RT^2^ Profiler™ PCR Array Human Osteogenesis (Qiagen Catalog #PAHS‐026ZD‐12) was used to assess the expression of 84 genes associated with osteogenesis. The cDNA was synthesized using the RT^2^ Profiler™ PCR Array protocol. Genes that were ±2‐fold change were validated with multiple samples. The iScript cDNA Synthesis Kit and protocol (Bio‐Rad Catalog Number 1708891) was used to generate cDNA from 500 ng of total RNA. Real‐time PCR reactions were performed using the SsoAdvanced Universal SYBR Green Supermix (Bio‐Rad Catalog Number 1725272) on a CFX QPCR instrument (Bio‐Rad). The targets and primers used are listed in Figure S6B. The IGF1R primer that was used to validate the RT_2_ Profiler™ blastic‐like cell line results was purchased from Qiagen (GeneGlobe). All genes were run in technical and biological quadruplicate, with glyceraldehyde 3‐phosphate dehydrogenase (GAPDH) as the housekeeping gene to normalize gene expression.

### 2.3 Statistics

Statistical analyses were performed using GraphPad Prism (version 9.4 for Windows; GraphPad Software Inc.) and Excel (version 2016 for Windows; Microsoft Corp.). All statistical tests used a cutoff *p* value of .05 for significance and were one‐tailed Mann–Whitney Students *t* test with assumed heteroscedasticity (non‐parametric).

### 2.4 cBioPortal analysis of the cancer genome atlas

cBioPortal.org datasets were used for the cancer genome atlas (TCGA) bioinformatics analysis. Prostate cancer data sets were selected for the adenocarcinoma study comprised in the firehose legacy data set.^27^ Metastatic prostate cancer samples were analyzed from the Stand‐Up‐To‐Cancer (SU2C) dataset by selecting groups from tissues' source locations (bone lymph node, liver, etc.).^28^ Cell line data from the CCLE Broad institute profiling were selected for prostate cancer cell lines and then queried for gene expression and responses to pharmacologic treatments.^23^ All samples' gene expression was compared to diploid samples with a ±Z score of 2.0. The cbioportal.org was used as a preferred curated resource of the TCGA datasets for robust harmonized uniformed analyses of prostate cancers.^21^, ^22^

## 3 RESULTS

### 3.1 Differential BMP pathway activity in prostate cancer adenocarcinomas and metastatic adenocarcinomas

**FIGURE 1:**
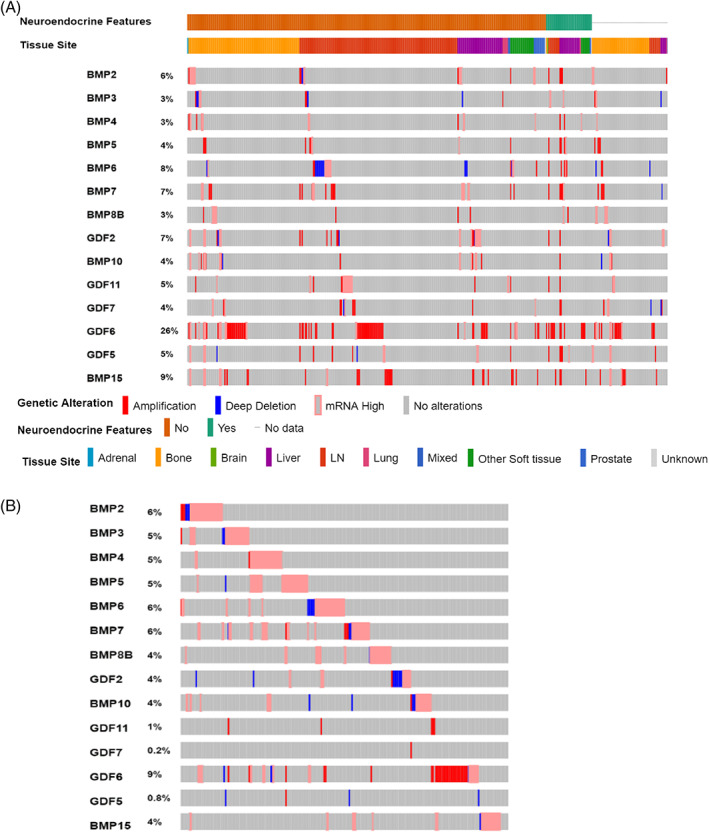
Bone morphogenetic protein (BMP) ligands are differentially expressed in metastatic prostate cancer and primary prostate adenocarcinomas. Metastatic (444 patients) and primary tumors (501 patients) from prostate are profiled in two separate studies available via the cancer genome atlas (TCGA) and accessed through the cBIO.org portal. Oncoprint graphs indicate whether a specific gene is amplified (red) or increased (pink) within the study or deleted (blue) or decreased (light blue). The percent of patients with any modification is indicated to the left of the graph following the gene name. (A) Ligands of the BMP pathway are listed in canonical order in metastatic prostate cancers and (B) can be observed in primary prostate adenocarcinomas. The metastatic data set allows for the additional tracks of neuroendocrine features as well as tissue specific sites of metastases for comparative analyses. Primary tumors are derived from the TCGA legacy firehose. Genomic profiles of mutations, copy number and mRNA expression relative to diploid samples RNA seq with a z‐score threshold of 2.0 were used in analysis

With the availability of diverse molecular genomic data available to appreciate the nature or molecular players in prostate cancer, we accessed TCGA via the cBioPortal. The BMP pathway is a large collection of secreted ligand, soluble antagonists and receptor signaling partners, which we simplified into these three respective categories—ligands, antagonists, and receptor/mediators (Figure S6A). We utilized the primary NCBI gene symbol identification for these genes and first queried ligands in primary prostate cancer adenocarcinomas. Unlike most cancers, prostate cancer benefits from also having TCGA data derived from metastatic tissues containing DNA, RNA, and protein changes with clinical outcomes for 444 patients. We queried the SU2C supported data set of metastatic prostate cancer (containing 444 patient samples) and first sorted and ordered the patients by neuroendocrine features and then the anatomic tissue source of the metastatic cancer. Neuroendocrine is of importance in the bone metastatic phenotype as these can be preferentially osteolytic than blastic.^17^, ^29^ The amplification of GDF6 seen in primary tumors was elevated to 26% of all metastatic patients followed next by BMP15 at 9% of patients (Figure 1B). We found that BMP ligands were more often overexpressed than lost or decreased and that their expression could be largely mutually exclusive from one ligand to the other. GDF6, also known as BMP‐13, was the highest expressed ligand at 9% of patients with primary prostate cancer followed by BMP 2, 6, and 7 at 6% of patients, with all other ligands being expressed less than 5% of patients (Figure 1B). Negative soluble secreted antagonists of BMP signaling displayed a much more diverse increase and decrease, with NOGGIN being consistently amplified and overexpressed in 6% of primary adenocarcinoma patients (Figure S1B). Interestingly, other BMP antagonists such as GREM1 and GREM2 have mixed genomic loss and increased RNA expression. While SOST and FST were more frequently lost, potentially synergizing with ligand increase (Figure S1B). Unlike ligands, soluble antagonists in metastatic prostate cancer patients are consistently increased in patients with CHRD at 12%, NOGGIN, SOSTDC1, FSTL1 at 7% and GREM2 at 6% of patients (Figure S1A). BMP receptors and their canonical signaling mediators displayed a unique mixture of genomic loss and increased gene expression (Figure S1C). BMP specific mediator SMAD9 was most strikingly lost with 15% of patients having unique alterations (Figure S1D). The alterations of SMDA9 were still present at 10% of metastatic patients (Figure S1C). BMPR1A in metastatic patients was altered at 10% but comprised a diverse set of alterations from amplification, deletion, increased and decreased gene expression (Figure S1C).

### 3.2 Distinct BMP pathway expression in human prostate cancer cell lines and response to therapeutics

**FIGURE 2:**
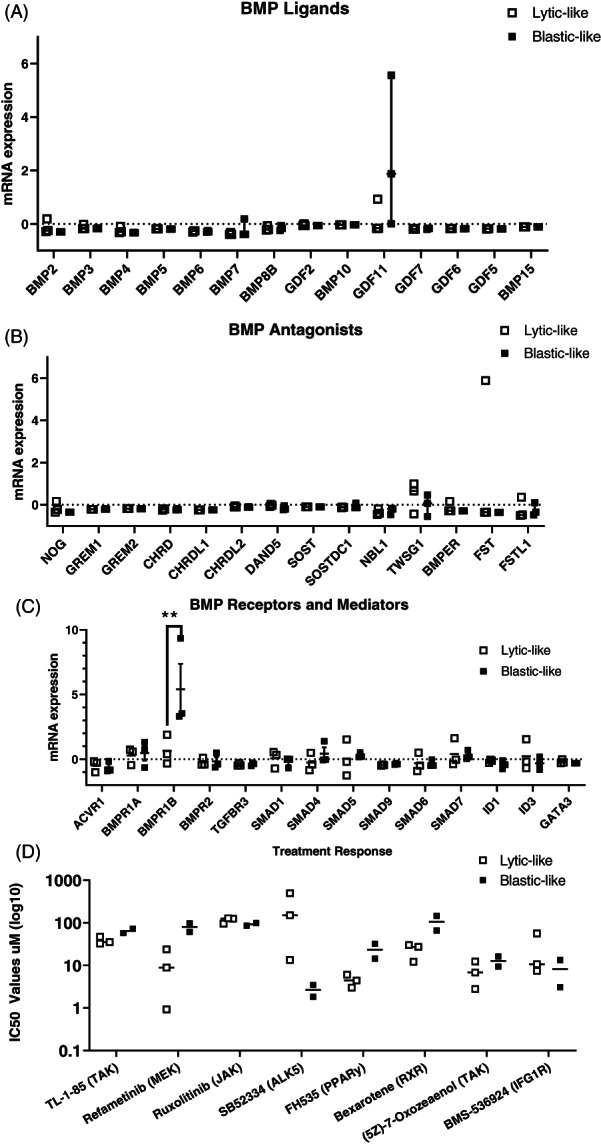
Distinct bone morphogenetic protein (BMP) pathway expression in human prostate cancer cell lines and response to therapeutics. The cancer genome atlas (TCGA) Cancer Cell Line Encyclopedia (CCLE) 2019 database contains sequencing, gene expression and treatment response data for human prostate cancer cell lines. Lytic‐like cell lines are comprised of PC3, 22Rv1 and Du‐145 lines. Blastic like cell lines are comprised of LNCaP, VCaP and MDA‐PCa‐2B lines. Treatment responses are not available for the MDA‐PCa‐2B cell line. (A) BMP ligands gene expression in lytic‐like (white boxes) and blastic‐like (black boxes) human prostate cancer cell lines. (B) BMP soluble ligand antagonists gene expression in human prostate cancer cell lines. (C) BMP receptors and intracellular mediators of signaling gene expression in human prostate cancer cell lines. (D) Select IC50 treatment responses to lytic‐like (*n* = 3) and blastic‐like (*n* = 2) human prostate cancer cell lines

The CCLE forever changed cancer research as hypotheses about mutations, transcriptomics, and proteomics in cancer cell lines could be directly investigated by anyone in silico.^21^ In 2019 the CCLE was amended with more cell lines and their gene expression changes in response to a large bevy of chemotherapeutic agents.^23^ Responses to pharmacologic agents are indicated by IC_50_ values, which indicate a therapeutic agent's half‐maximal inhibitory concentration to reduce Cell Titer Glo cell viability. Within the CCLE, we selected the lytic‐like cell lines PC3, 22Rv1, and Du145 in one group and the blastic‐like cell lines LNCaP, VCaP, and MDA‐PCa‐2b in another group to compare BMP signaling components (Figure 2). When lytic‐ and blastic‐like human prostate cancer cell are grown alone (potentially requiring a true microenvironment) in tissue culture they do not display any significant BMP ligand gene expression changes between these groups apart from *GDF11* elevated in the blastic‐like cells (Figure 2A). Soluble antagonists to BMP signaling were also unremarkable in comparison to lytic‐ or blastic‐like tumor induced bone disease phenotype prostate cancer cell lines, with only *TWSG1* having slight elevation in lytic‐like cells (Figure 2B). For BMP receptors and their signaling surrogates an interesting pattern of *BMPR1B* gene expression was elevated in blastic‐like cell lines with downstream mediators largely similar across all cell lines (Figure 2C). When lytic‐ and blastic‐like cell lines were compared for their IC_50_ responses^23^ to different targeted agents it was observed that lytic‐like cell lines were more sensitive and had lower IC_50_ concentrations for the MEK pathway inhibitor Refametinib and PPARy inhibitor FH535 (Figure 2D). Blastic‐like cell lines were more sensitive and had lower IC_50_ values for the ALK5 TGFβ inhibitor SB52334, indicating not BMP but TGFβ receptor signaling to be more dynamic in blastic‐like cell types.

### 3.3 The effect of BMP inhibition on human blastic prostate cancer cell line osteogenesis gene expression

**FIGURE 3:**
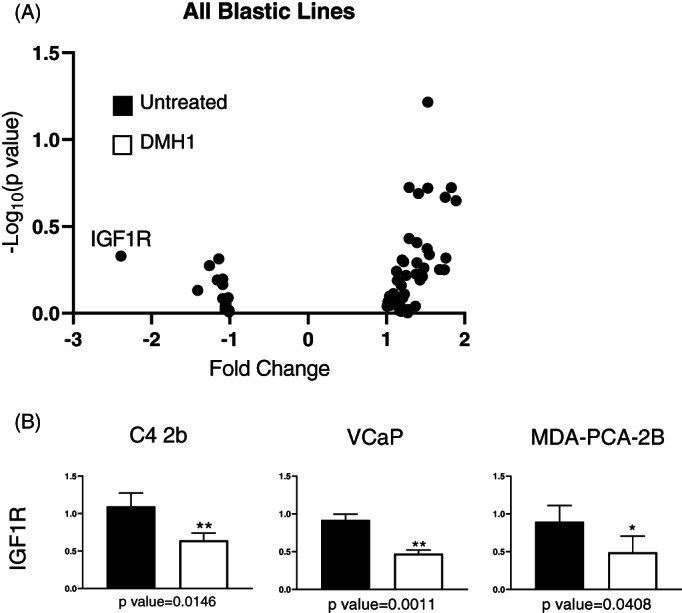
The effect of bone morphogenetic protein (BMP) inhibition on human blastic‐like prostate cancer cell lines for osteogenesis gene expression. Blastic‐like prostate cancer cell lines were treated with BMP type I receptor inhibitor DMH1 at 10 μM for 24 h. RNA was isolated and an RT^2^ Profiler PCR Array Human Osteogenesis was run for three prostate cancer lines. The DMH1 sample was compared to an untreated sample (DMSO treated). (A) Gene expression for osteogenesis genes were analyzed all together to see which genes were changed 2‐fold. IGF1R was decreased 2‐fold. (B) Validation of IGF1R expression was confirmed separately by standard SYBR qPCR in all three blastic‐like cell lines C4‐2b, VCaP and MDA‐Pca‐2b human prostate cancer cells

With BMP ligands upregulated in both primary and metastatic prostate cancers, we chose to test a collection of human prostate cancer cell lines with known blastic bone phenotypes potentially stimulated by BMP activation and driving osteogenesis.^30^ The VCAP, C4‐2b, and MDA‐PCa‐2B cell line were chosen as blastic‐like cell lines and treated with the small molecule type I BMP receptor kinase antagonist DMH1 at 10 μM for 24 h. We specifically employed a focused 84‐gene panel of commercially developed Human Osteogenesis PCR array to test this BMP driving osteogenesis that could be inhibited by BMP inhibition. While DMH1 has been shown to be a selective and specific type 1 BMP receptor antagonists it still requires micro molar levels of efficacy making in vivo use difficult and relegating its use as an in vitro research reagent.^31^, ^32^ To validate the canonical BMP response, we measured the impact of DMH1 BMP signaling inhibition on *ID1*, *SMAD6*, and *SMAD7* gene expression without BMP stimulation. We found that gene expression of *ID1*, a downstream target gene of canonical BMP signaling, was significantly reduced in the DMH1 group of all three cell lines (Figure S2A). While response genes *SMAD6* and *SMAD7* exhibited, only moderate gene expression inhibition upon DMH1 treatment. BMP inhibition in VCAP cells led decreased gene expression of osteogenesis genes *IGF1R*, *TGFB2*, *COL10A1* and *BMPR1B* (Figure S3A). Genes increased in these conditions were *SP7* (also known as osterix), *FN1*, and *CTSK*. C4‐2b cells also resulted in reduced transcription of *IGF1R* as well as *SOX9*, while a transcriptional increase of *BMP6* was observed when BMP was inhibited with DMH1 (Figure S3A). MDA‐PCa‐2B cells treated with DMH1 consistently resulted in *IGF1R* reduction along with decreased in *IGF1* gene expression (Figure S3B). Several genes were increased with BMP inhibition in MDA‐PCa‐2b cells, including *SOX9*, *MMP10*, *ITGTA1*, *COL3A1*, *COL2A1*, and *BMP3* (Figure S3C). For all three blastic‐like cell lines, the only consistent osteogenesis related factor which exhibited altered gene expression upon DMH1 treatment was the decrease of *IG1R* (Figure 3A). Additional validation of *IGF1R* mRNA reduction was done by commercially validated qPCR primer set and again resulted in statistically significant *IGF1R* mRNA loss in blastic‐like cell lines C4‐2b, VCAP, and MDA‐PCa‐2B (Figure 3B).

### 3.4 The effect of BMP inhibition on human lytic prostate cancer cell lines osteogenesis transcripts

**FIGURE 4:**
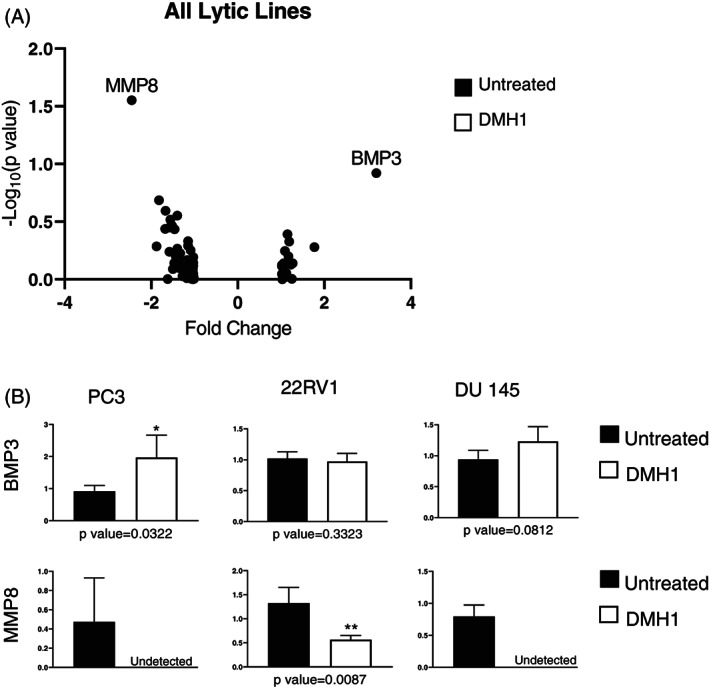
The effect of bone morphogenetic protein (BMP) inhibition on human lytic‐like prostate cancer cell lines for osteogenesis gene expression. Lytic‐like prostate cancer cell lines were treated with BMPR1a inhibitor DMH1 for 24 h. RNA was isolated and an RT^2^ Profiler PCR Array Human Osteogenesis was run for three prostate cancer lines. The DMH1 sample was compared to an untreated sample. (A) Gene expression for osteogenesis genes were analyzed all together to see which genes were changed 2‐fold. MMP8 was decreased 2‐fold, while BMP3 was increased 2‐fold. (B) Validation of MMP8 and BMP3 expression was confirmed separately by standard SYBR qPCR in all three lytic‐like cell lines PC3, 22Rv1 and Du‐145 human prostate cancer cells

With BMP's complex roles in both osteogenesis and other developmental signaling pathways, we next sought to see the response of BMP inhibition on prostate cancer cell lines that are known to exhibit osteolytic bone disease. The Du145, PC3, and 22Rv1 human prostate cancer cells were chosen for their previously exhibited lytic functions in experimental bone metastases.^30^ These three cell lines were treated with DMH1 at 10uM for 24 h to block endogenous BMP signaling. To assess the reduction in canonical BMP signaling, we again measured *ID1*, *SMAD6*, and *SMAD7* gene expression as surrogates of BMP activity. We found that all three cell lines had significantly reduced gene expression of *ID1* and *SMAD6* upon DMH1 treatment (Figure S2B). *SMAD7* was not found to be affected in PC3 cells but was reduced in 22Rv1 and Du145 cells with BMP inhibition. *SMAD7* is known to be mutated and exhibit distinct functions in PC3 cells and is a downstream mediator of the entire TGFβ signaling family not exclusive to BMP activity. BMP inhibition of PC3 cells also resulted in gene expression increases in BMP3 and reduction of MMP8 (Figure S4A). PC3 cells inhibited by DMH1 also saw decreased gene expression for *FLT1* and *VDR*. Interestingly, *CSF3* gene expression was increased by more than 4‐fold in response to BMP inhibition in PC3 cells, which was the opposite trend observed in Du145 cells. 22Rv1 cells when treated with BMP inhibitor DMH1 once again showed increase *BMP3* and decreased *MMP8* mRNA (Figure S4B). 22Rv1 cells also saw loss of *TGFB1*, *IGF1R*, *IGF1*, and *BMP6* with increased *EGF* gene expression. BMP inhibition in Du145 cells resulted in osteogenesis genes expression decreases for *MMP8*, *NOG*, *MMP10*, *GLI1*, and *CSF3* (Figure S4C). Genes increased by BMP inhibition in Du145 cells included *BMP3* and *CSF2*. Across all three lytic‐like human prostate cancer cell lines, BMP inhibition resulted in decreased *MMP8* and increased *BMP3* gene expression (Figure 4A). Additional qPCR validation with additional primers only partially confirmed these results; however, 22Rv1 and Du145 cells did not show significant *BMP3* elevation (Figure 4B). The decreased *MMP8* mRNA was challenging to analyze as only 22Rv1 cells had detectable levels of mRNA to quantitate under DMH1 treatment, while PC3 and Du145 cells *MMP8* was not detected following BMP inhibition (Figure 4B).

### 3.5 Distinct mutations and BMP pathway gene expression in metastatic tissue sites of prostate cancer

**FIGURE 5:**
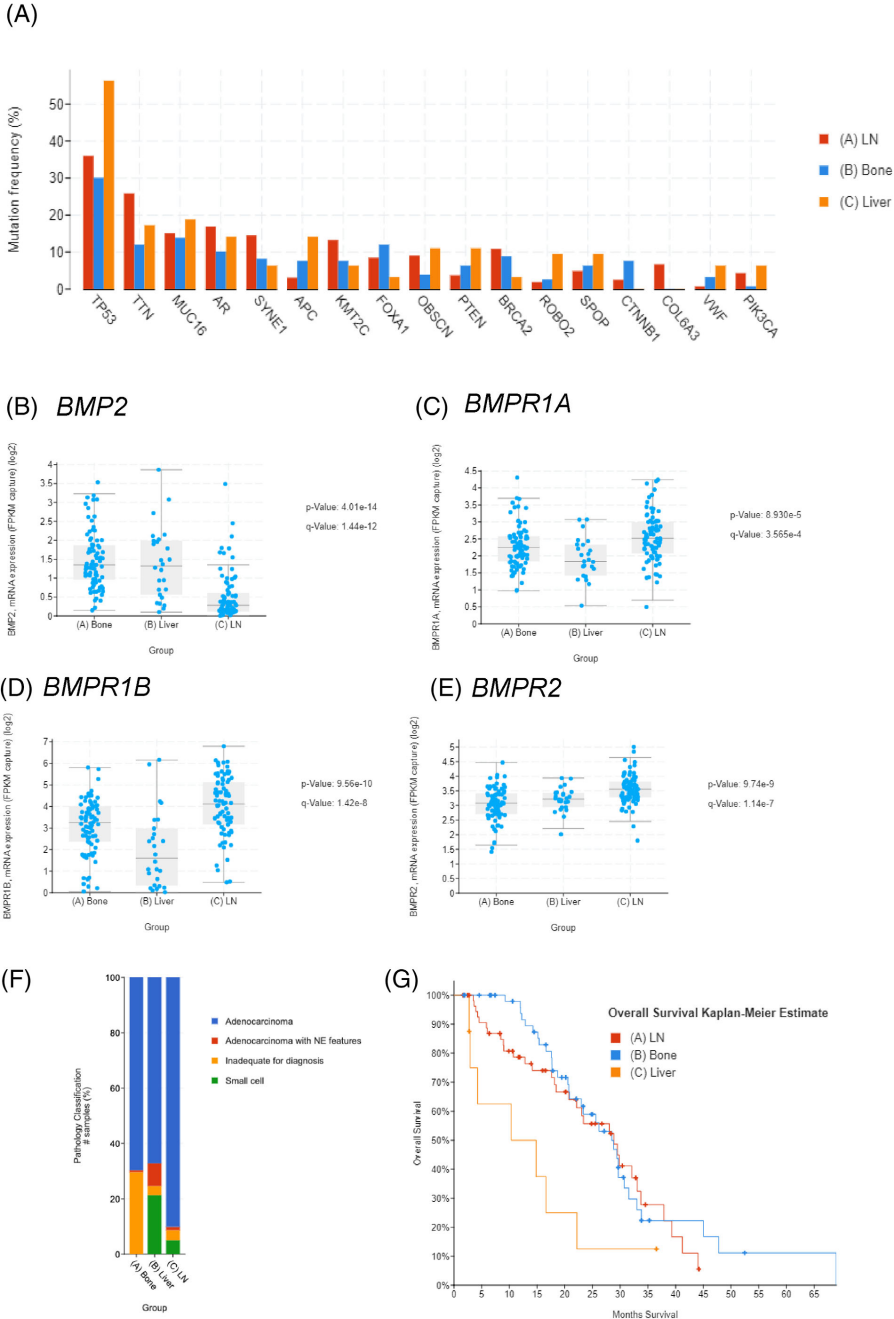
Metastatic tissue sites of prostate cancer display distinct mutations, gene expression and bone morphogenetic protein (BMP) expression. The cBIO portal was used to compare the 2019 PNAS metastatic prostate cancer data set by tissue site. (A) Mutation frequencies are largely expected but demonstrate that not all mutation frequencies occur at all metastatic sites. All patients were mutually exclusive and not represented in more than one group. (B) Gene expression of BMP2 mRNA demonstrated specific enrichment for the host tissue site of the bone metastasis. (C–E) BMPR1A, BMPR1B and BMPR2 canonical BMP receptors were significantly elevated in the lymph node (163 unique patients) prostate cancer metastases compared to liver (63 patients) and bone (152 patients). (F) The composition of bone, lymph node and liver metastasis were primarily adenocarcinoma with the liver having the highest concentration of tumors with neuroendocrine‐like features. (G) Overall survival at distinct tissue sites demonstrate enhanced survival for lymph node and bone metastases compared with liver metastases

Prostate cancer is unique in that the metastatic sites of disease beyond the primary site have been investigated when many other cancers with metastasis still rely on data from the primary tumor. The 2019 Su2C prostate metastasis study is unique in that it provides insight into lymph node, bone, and liver metastases. We decided to analyze the prostate cancer by metastatic location in bone (152 unique patients), lymph node (163 unique patients) and liver (63 unique patients) groups, which were non‐overlapping to determine first if their mutational landscapes were distinct. We found that the most commonly mutated genes were similar across the three sites such as p53 and AR (Figure 5A). Gene expression across these three tissues revealed that BMP2 ligand was lowest in lymph nodes and elevated in bone and liver and receptors BMPR1A and BMPR1B were highest in lymph node metastases (Figure 5B–D). While the type II receptor BMPR2 was broadly expressed across all tissues (Figure 5E). The majority of these metastatic prostate cancers were of adenocarcinoma with little representing the neuroendocrine phenotype (Figure 5F). When survival was analyzed by disease location a clear separation of these patients could be seen for superior survival of lymph node and bone only metastatic disease when compared with liver metastases (Figure 5G). These results suggest that location of metastasis may be correlated with mutations and gene expression within the BMP signaling pathway from either the tumor or its unique tissue microenvironment. Copy number alterations separated by tissue location revealed subtle reductions in applications for the liver disease (Figure S5A). Looking at the most disparate amplifications or deletions resulted in distinct changes in liver disease such as RB1 deletions occurring at 25% of patients with little to no mutations were found in bone and just over 10% were found in lymph node (Figure S5B). When metastatic prostate cancer is separated by the location of the disease a clear pattern of distinct genetic profiles emerges that can be successfully distinguished and diagnosed.

## 4 DISCUSSION

When Dr Marshall Urist first discovered the soluble factors to be known as BMPs, it set in motion a fundamental quest for secreted growth factors capable of determining cell fates.^33^ As prostate cancer demonstrates strong tropism to bone and uniquely generates blastic, sclerotic, and excess mineralized matrix it becomes logical to associate this presentation with the osteoinduction by BMPs that Dr Urist has observed. The quest to investigate BMP expression in prostate cancer was enabled by molecular and histological advances that allowed for specific BMPs to be identified in prostate cancer.^13^ Additional work confirmed a myriad of dysregulated BMPs in various prostate cells, tissues and clinical samples.^34^, ^35^, ^36^, ^37^, ^38^, ^39^With the advent of the TCGA studies in prostate cancers, the ability to query any signaling pathway was made available.^27^ Our findings in this study shed light on the genetic and transcriptional changes for the BMP pathway in prostate cancers and their subsequent metastases. Future studies showing single cell and spatial transcriptomics/proteomics will continue to enhance our precision understanding of not only metastatic prostate cancer but many other diseases.^26^ Molecular and pathological precision of rarer subtypes of prostate cancer, such as neuroendocrine phenotypes are already well under way.^40^ The framework for diagnosis and prospective treatments in prostate cancer have already been described, which also demonstrated the increased risk of genomic loss of BMPR1a enriched in metastatic castrate resistant prostate cancer.^41^

Prostate cancer's osteo‐tropism is unique for its predilection to the blastic phenotype whereby bone is not lost from a vicious cycle of osteoclast bone destruction but instead promotes a poorly organized woven matrix of mineralized collagen. BMP driving osteogenesis has been proposed to drive this blastic phenotype and BMP inhibition was hypothesized to reduce the osteogenesis program (Figure 3). In three human blastic‐like prostate cancer cell lines we showed not a reduction in osteogenesis, but a convergence with the IGF‐IGF1R signaling axis (Figure 3A). IGF1R is currently being investigated as a potential biomarker in metastatic prostate cancers, as appropriate biomarkers that may be derived in the blastic bone phenotype have not yet been identified.^42^ Targeting the IGF pathway in tumor induced bone disease has shown great promise alone or in therapeutic combinations. Yet the roles of IGF and BMPs in skeletal health require further investigation to understand the best patient‐precise therapeutic interventions.^43^ The osteomimicry seen by BMPs has been demonstrated previously in the conditioned medium or co‐culture of osteoblasts with prostate cancer cells.^16^, ^19^, ^20^, ^44^, ^45^, ^46^, ^47^ These studies all demonstrate the sufficiency of prostate cancer cell lines to secrete BMPs to promote an osteogenic program, yet inhibition of BMP may require further coordination of additional signaling pathways.^48^

There remain unresolved mechanisms for the formation of the blastic lesion or the maintenance of the mixed lesion containing both the blastic scleroses and evidence of heightened resorption in lytic lesions. We were surprised by the lack of transcriptional osteogenesis in blastic‐like cell lines treated with BMP inhibition, thus we turned to compare lytic‐like cell lines for osteogenesis under BMP inhibition (Figure 3). BMPs have recently been shown to have expanded roles in osteoclasts and bone resorption which can be instrumental in the heightened lytic vicious cycle of tumor induced bone disease.^49^, ^50^ BMP inhibition of lytic‐like lesions may be more preferential than blastic‐like lesions if those lesions are driven by BMP‐directed osteoclastogenesis as compared to TGFβ‐driven sclerosis.^51^ It has also been demonstrated in multiple myeloma that BMP inhibition of osteolytic lesions improved bone quality through combined lineages of bone matrix producing cells.^52^ Genetic and pharmacologic loss of function studies for BMPR1a have surprisingly resulted in improvement in bone formation as opposed to expected loss in BMP directed osteogenesis.^53^, ^54^, ^55^ We found that BMP inhibition in lytic‐like prostate cancer cell lines increases the transcription of BMP3 in some of the cell lines (Figure 4B). BMP3 is not well studied in comparison to with more osteogenic BMPs 2/4/7 or even BMP6 and has unique negative functions in skeletal biology.^56^, ^57^ This alternative BMP3 response could explain the many ways that prostate cancer induced bone disease creates a unique bone matrix environment that is not a full recapitulation of a healthy osteogenesis program. MMP8 transcription was also found to be regulated and lost upon BMP inhibition in lytic‐like prostate cancer cells, suggesting that lytic‐like cells may use MMP8 to facilitate the destruction of bone with matrix degradation.^58^ MMP8 is also unique and interesting as a putative metastasis suppressor, yet the role of MMP8 in prostate cancer metastasis in bone remain to be fully elucidated.^59^ MMP8, like TGFβ/BMP, has many nuanced and context‐dependent roles that may be both tumor promoting and or suppressive, a context that may be important to the distinct process a tumor cell is attempting to achieve in a specific microenvironment.^60^ It is unsurprising that the BMP pathway is not largely distinguished for in vitro gene expression (Figure 2). However, the finding that BMPR1b and not BMPR1a is elevated in blastic‐like prostate cancer cell lines is compelling (Figure 2C). One of the most compelling BMP receptor studies has shown that BMPR1b to be uniquely induced by androgen unlike the other type 1 receptors.^61^ Blastic‐like cell lines are more commonly androgen sensitive to both stimulation and deprivation which raises unanswered questions about the blastic matrix phenotype and androgen.^30^

Metastatic prostate cancer has been a pioneer for metastatic disease research as numerous patients and their progression have provided scientists with unique datasets for in silico analysis such as the TCGA datasets.^28^ Our finding of specific genetic forms of metastatic prostate cancer independent of tumor location is compelling for the development of precise treatments based on genotype. However prognostic markers enriched in different tissue sites, such as BMPs demonstrated in this study, highlight the unique nature of metastatic prostate cancers microenvironments. Many metastatic prostate cancer patients have oligometastatic disease, thus precision oncology treatment regimens may benefit from tissue‐specific prognostic indicators. Multiple organ burden of metastatic disease with multiple genomic and non‐genetic mechanisms of disease remains a penultimate challenge for cancer treatment, where diverse systemic heterogenous disease may be difficult to have a single precise or accurate target for therapeutic intervention.

## 5 LIMITATIONS OF THE STUDY

A major limitation of this study is the non‐cell autonomous functions of BMPs. BMPs are used in tissues to coordinate diverse cell behavior and these studies examine BMP inhibition of human prostate cancer cells grown on tissue culture plastic. Additionally the observations made by BMP mRNA in TCGA data sets are not fully representative of BMP signaling via proteomic and direct signaling observations. These TCGA data sets are useful in that they are one of the only that show distinct metastatic sites but unfortunately are not annotated with the clinical diagnosis of bone disease being osteoblastic or osteolytic. Prostate cancer cell lines are limited to less than a dozen models, which may not faithfully represent the full spectrum of disease or include a diverse representation of the many men who suffer from the disease. While the generation of new human and mouse models of prostate cancer continues, these resources should be expanded to further enhance the representation of the metastatic disease and its diverse disease progression in response to frontline therapies to improve future research studies.

## AUTHOR CONTRIBUTIONS

**Meredith D. Provera:** Conceptualization (lead); data curation (lead); formal analysis (equal); methodology (equal); writing – original draft (equal); writing – review and editing (equal). **Desiree M. Straign:** Data curation (equal); methodology (equal); supervision (equal); validation (equal); writing – review and editing (equal). **Parvanee Karimpour:** Data curation (equal); investigation (equal); writing – original draft (equal). **Claire L. Ihle:** Conceptualization (equal); formal analysis (equal); investigation (equal); writing – original draft (equal); writing – review and editing (equal). **Philip Owens:** Conceptualization (equal); data curation (equal); formal analysis (equal); funding acquisition (lead); investigation (equal); methodology (equal); project administration (lead); writing – original draft (equal); writing – review and editing (lead).

## CONFLICT OF INTEREST

The authors have stated explicitly that there are no conflicts of interest in connection with this article.

## ETHICS STATEMENT

All research was exempt from IRB as all human subjects were not identified and data was from publically available repostitories.

## Supporting information


**FIGURE S1 The BMP pathway regulators and effectors are differentially active in prostate cancer metastatic adenocarcinomas and primary adenocarcinomas.** Metastatic (444 patients) and primary tumors (501 patients) from prostate are profiled in two separate studies available at the TCGA and accessed through the cBIO.org portal. Oncoprint graphs indicate whether a specific gene is amplified (red) or increased (pink) within the study or deleted (blue) or decreased (light blue). The percent of patients with any modification is indicated to the left of the graph following the gene name. (A) Soluble BMP ligand antagonist expression in (B) Adenocarcinomas compared with (A) metastases. (C‐D) BMP signaling is mediated by receptors, SMAD intracellular proteins and canonically activate transcription of ID1, ID3 and GATA3 expression in metastatic and primary prostate tumors. Primary tumors are derived from the TCGA legacy firehouse. Genomic profiles of mutations, copy number and mRNA expression relative to diploid samples RNA seq with a z‐score threshold of 2.0. The SU2C metastatic data set allows for the additional tracks of neuroendocrine features as well as tissue specific sites of metastases for comparative analyses.
**FIGURE S2. Canonical BMP signaling inhibition by DMH1.** Prostate cancer cell lines were treated with BMPR1a inhibitor DMH1 for 24 hours at 10 μM concentration. Gene expression for BMP signaling read outs, ID1, SMAD6 and SMAD7 for (A) Blastic‐like and (B) Lytic‐like cell lines.
**FIGURE S3. The effect of BMP inhibition on human blastic‐like prostate cancer cell lines osteogenesis transcripts.** Blastic‐like prostate cancer cell lines were treated with BMP type I receptor inhibitor DMH1 at 10uM for 24 hours. RNA was isolated and an RT^2^ Profiler PCR Array Human Osteogenesis was run for three prostate cancer lines. The DMH1 sample was compared to an untreated sample (DMSO treated). (A) Genes were increased or decreased 2‐fold in C4 2b cell line. (B) Genes that were increased or decreased by 2‐fold in the VCaP cell line are shown as compared to the untreated group. (C) Genes were increased or decreased 2‐fold in MDA PCA 2B cell line.
**FIGURE S4. The effect of BMP inhibition on human lytic‐like prostate cancer cell lines osteogenesis transcripts.** Lytic‐like prostate cancer cell lines were treated with BMPR1a inhibitor DMH1 for 24 hours. RNA was isolated and an RT^2^ Profiler PCR Array Human Osteogenesis was run for three prostate cancer lines. The DMH1 sample was compared to an untreated sample. (A) Genes were increased or decreased 2‐fold in PC3 cell line. (B) Genes that were increased or decreased by 2‐fold in the 22Rv1 cell line are shown as compared to the untreated group. (C) Genes were increased or decreased 2‐fold in Du145 cell line.
**FIGURE S5. Distinct copy number alterations in metastatic prostate cancer by metastasis location.** The cBIO portal was used to compare the 2019 PNAS metastatic prostate cancer data set by tissue site. All patients were mutually exclusive and not represented in more than one group.
**FIGURE S6. BMP Pathway genes.** (A) The BMP pathway can be simplified into ligands, soluble antagonists and intracellular signaling components. (B) SYBR qPCR primers used at 60° to validate QIAGEN Osteogenesis qPCR arrays.

## Data Availability

Any and all data to support the conclusions will be available by the authors with no reservation or restriction.
