# Supplementary material for: Bone morphogenetic protein pathway responses and alterations of osteogenesis in metastatic prostate cancers
Source: Cancer Rep (Hoboken). 2022 Aug 19;6(2):e1707. doi: 10.1002/cnr2.1707 (PMC9940003; doi:10.1002/cnr2.1707)
Supplement: Supplementary file 1 — FIGURE S1 The BMP pathway regulators and effectors are differentially active in prostate cancer metastatic adenocarcinomas and primary adenocarcinomas. Metastatic (444 patients) and primary tumors (501 patients) from prostate are profiled in two separate studies available at the TCGA and accessed through the cBIO.org portal. Oncoprint graphs indicate whether a specific gene is amplified (red) or increased (pink) within the study or deleted (blue) or decreased (light blue). The percent of patients with any modification is indicated to the left of the graph following the gene name. (A) Soluble BMP ligand antagonist expression in (B) Adenocarcinomas compared with (A) metastases. (C‐D) BMP signaling is mediated by receptors, SMAD intracellular proteins and canonically activate transcription of ID1, ID3 and GATA3 expression in metastatic and primary prostate tumors. Primary tumors are derived from the TCGA legacy firehouse. Genomic profiles of mutations, copy number and mRNA expression relative to diploid samples RNA seq with a z‐score threshold of 2.0. The SU2C metastatic data set allows for the additional tracks of neuroendocrine features as well as tissue specific sites of metastases for comparative analyses. FIGURE S2. Canonical BMP signaling inhibition by DMH1. Prostate cancer cell lines were treated with BMPR1a inhibitor DMH1 for 24 hours at 10 μM concentration. Gene expression for BMP signaling read outs, ID1, SMAD6 and SMAD7 for (A) Blastic‐like and (B) Lytic‐like cell lines. FIGURE S3. The effect of BMP inhibition on human blastic‐like prostate cancer cell lines osteogenesis transcripts. Blastic‐like prostate cancer cell lines were treated with BMP type I receptor inhibitor DMH1 at 10uM for 24 hours. RNA was isolated and an RT2 Profiler PCR Array Human Osteogenesis was run for three prostate cancer lines. The DMH1 sample was compared to an untreated sample (DMSO treated). (A) Genes were increased or decreased 2‐fold in C4 2b cell line. (B) Genes that were increa [file CNR2-6-e1707-s001.pdf]

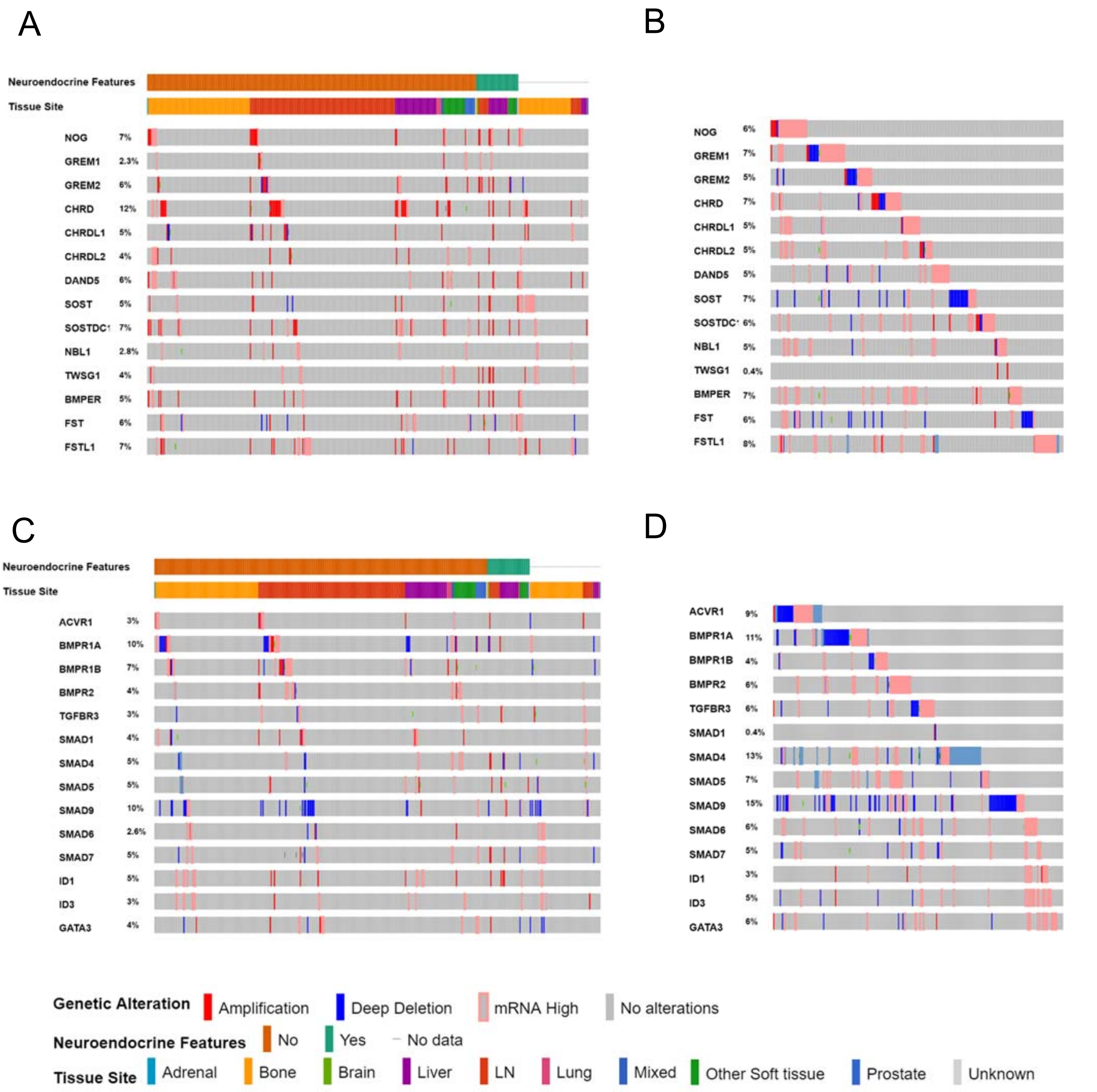

Supplemental Figure 1. The BMP pathway regulators and effectors are differentially active in prostate cancer metastatic adenocarcinomas and primary adenocarcinomas. Metastatic and primary tumors from prostate are profiled in two separate studies available at the TCGA and accessed through the cBIO.org portal. Oncoprint graphs indicate whether a specific gene is amplified (red) or increased (pink) within the study or deleted (blue) or decreased (light blue). The percent of patients with any modification is indicated to the left of the graph following the gene name. A) Soluble BMP ligand antagonist expression in B) Adenocarcinomas compared with A) metastases. C-D) BMP signaling is mediated by receptors, SMAD intracellular proteins and canonically activate transcription of ID1, ID3 and GATA3 expression in metastatic and primary prostate tumors. Primary tumors are derived from the TCGA legacy firehouse. Genomic profiles of mutations, copy number and mRNA expression relative to diploid samples RNA seq with a z-score threshold of 2.0. The SU2C metastatic data set allows for the additional tracks of neuroendocrine features as well as tissue specific sites of metastases for comparative analyses.

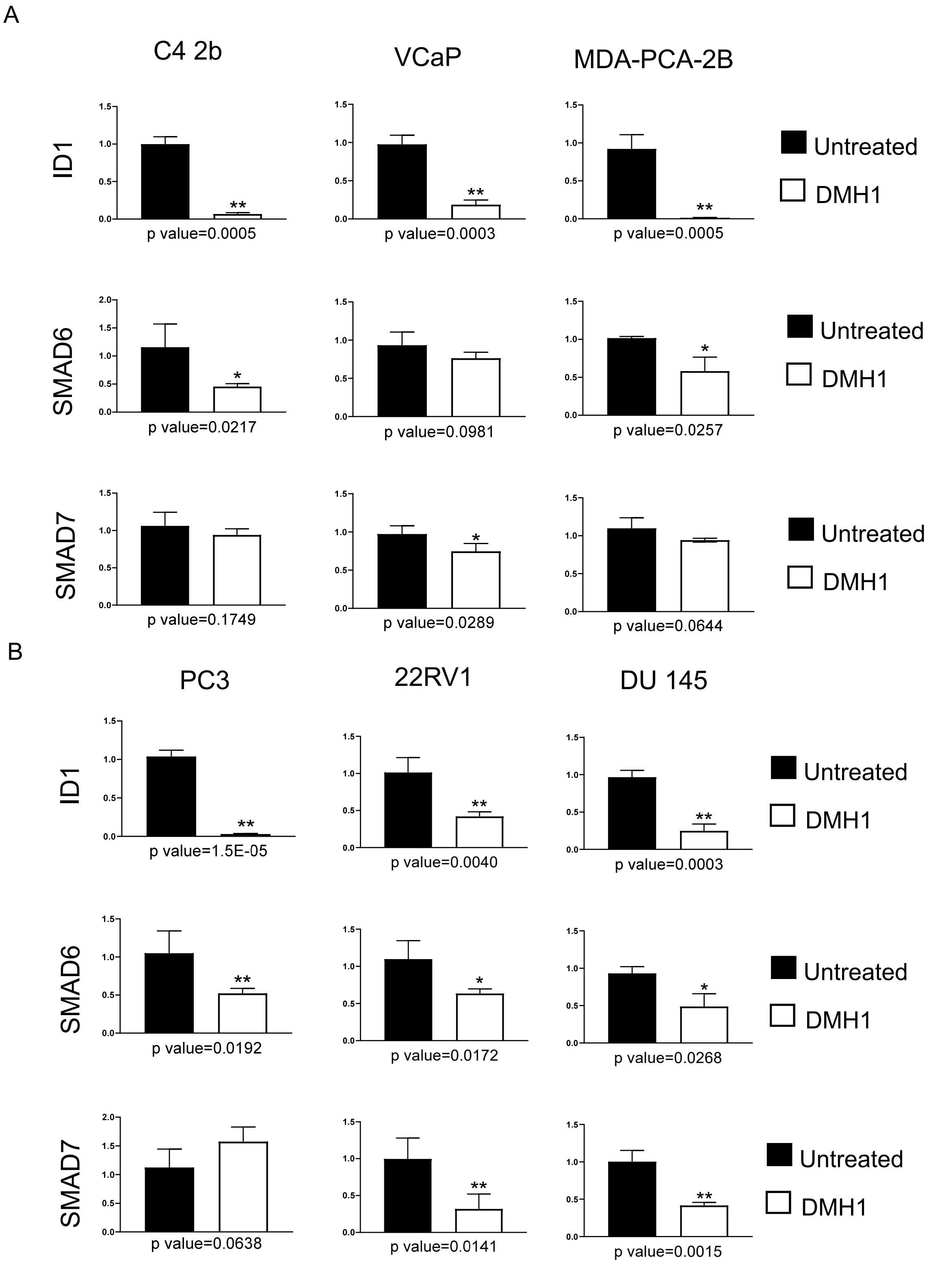

Supplemental Figure 2. Canonical BMP signaling inhibition by DMH1. Prostate cancer cell lines were treated with BMPR1a inhibitor DMH1 for 24 hours at 10μM concentration. Gene expression for BMP signaling read outs, ID1, SMAD6 and SMAD7 for A) Blastic-like and B) Lytic-like cell lines.

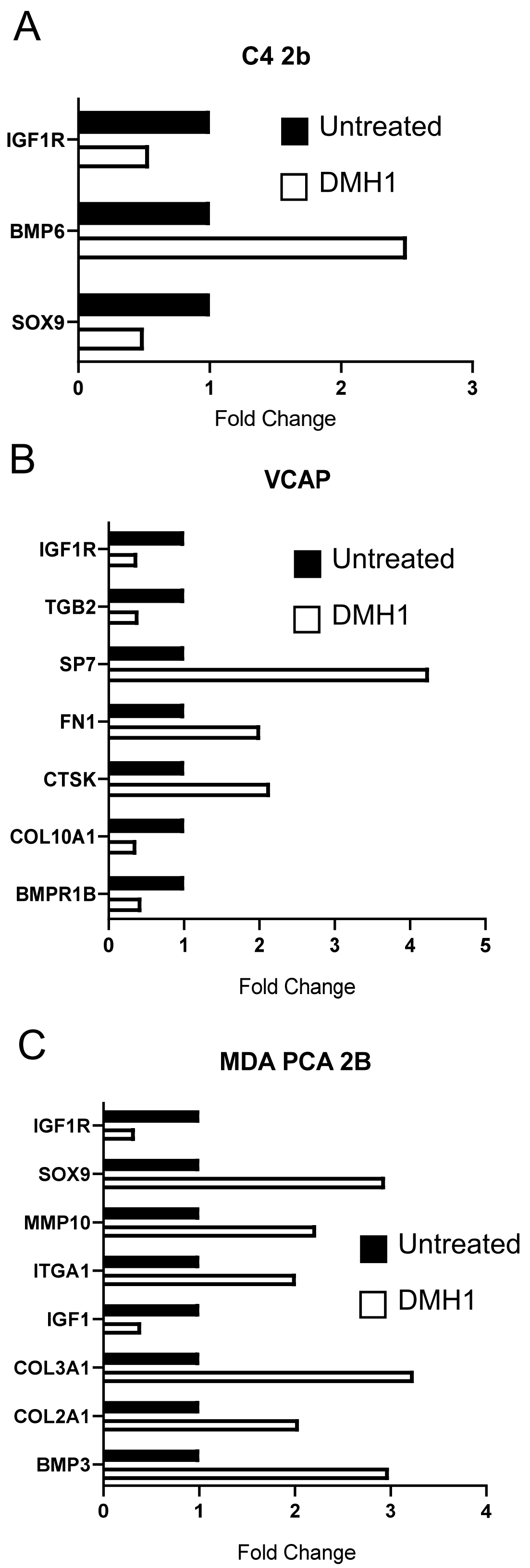

Supplemental Figure 3. The effect of BMP inhibition on human blastic-like prostate cancer cell lines osteogenesis transcripts. Blastic-like prostate cancer cell lines were treated with BMP type I receptor inhibitor DMH1 at 10uM for 24 hours. RNA was isolated and an RT^2 Profiler PCR Array Human Osteogenesis was run for three prostate cancer lines. The DMH1 sample was compared to an untreated sample (DMSO treated). A) Genes were increased or decreased 2-fold in C4 2b cell line. B) Genes that were increased or decreased by 2-fold in the VCaP cell line are shown as compared to the untreated group. C) Genes were increased or decreased 2-fold in MDA PCA 2B cell line.

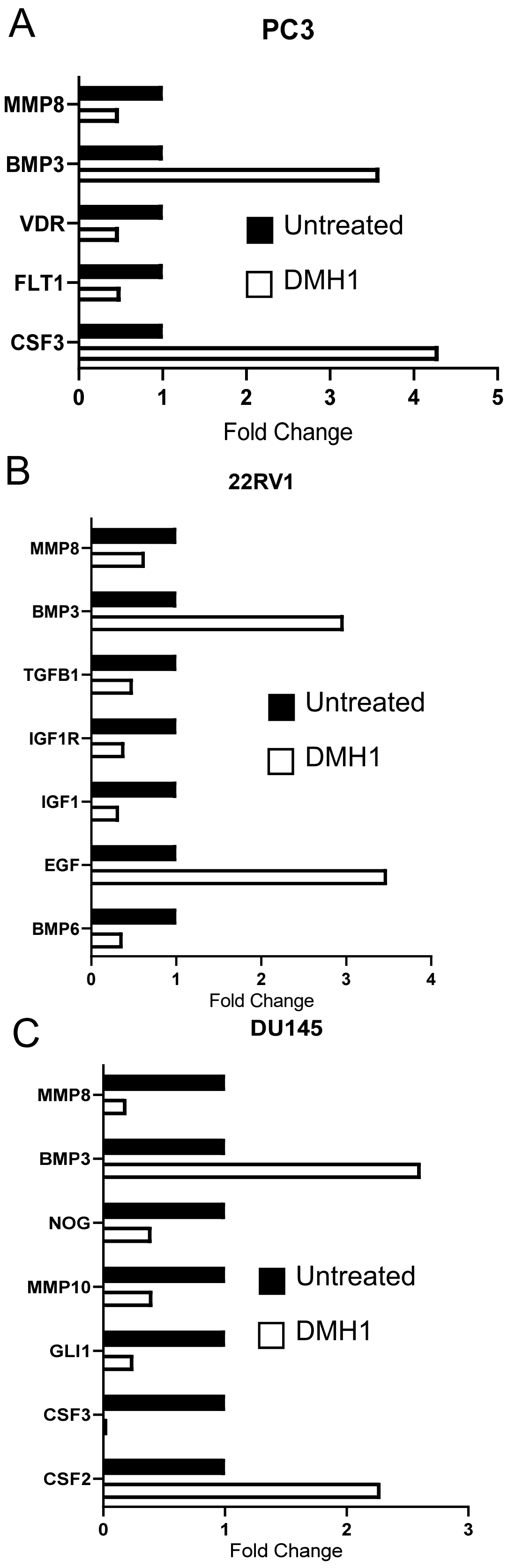

Supplemental Figure 4. The effect of BMP inhibition on human lytic-like prostate cancer cell lines osteogenesis transcripts. Lytic-like prostate cancer cell lines were treated with BMPR1a inhibitor DMH1 for 24 hours. RNA was isolated and an RT^2 Profiler PCR Array Human Osteogenesis was run for three prostate cancer lines. The DMH1 sample was compared to an untreated sample. A) Genes were increased or decreased 2-fold in PC3 cell line. B) Genes that were increased or decreased by 2-fold in the 22Rv1 cell line are shown as compared to the untreated group. C) Genes were increased or decreased 2-fold in Du145 cell line.

A

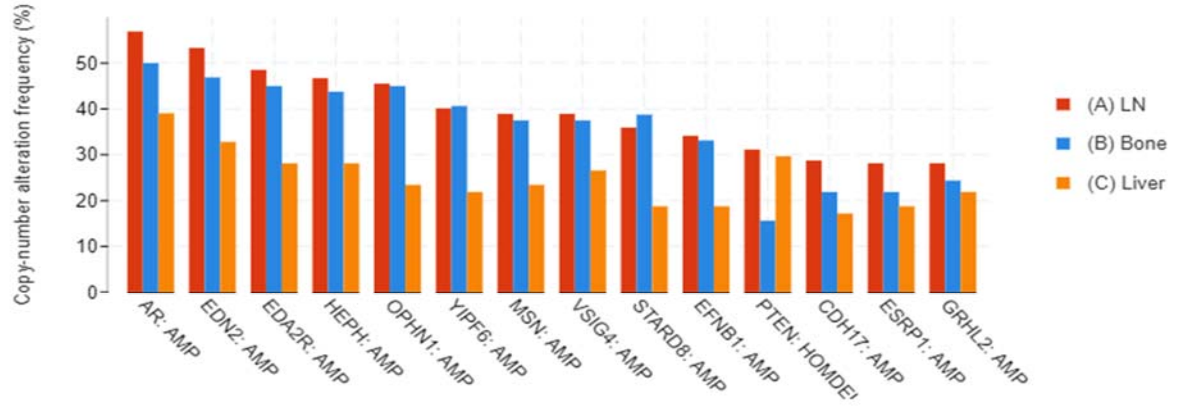

B

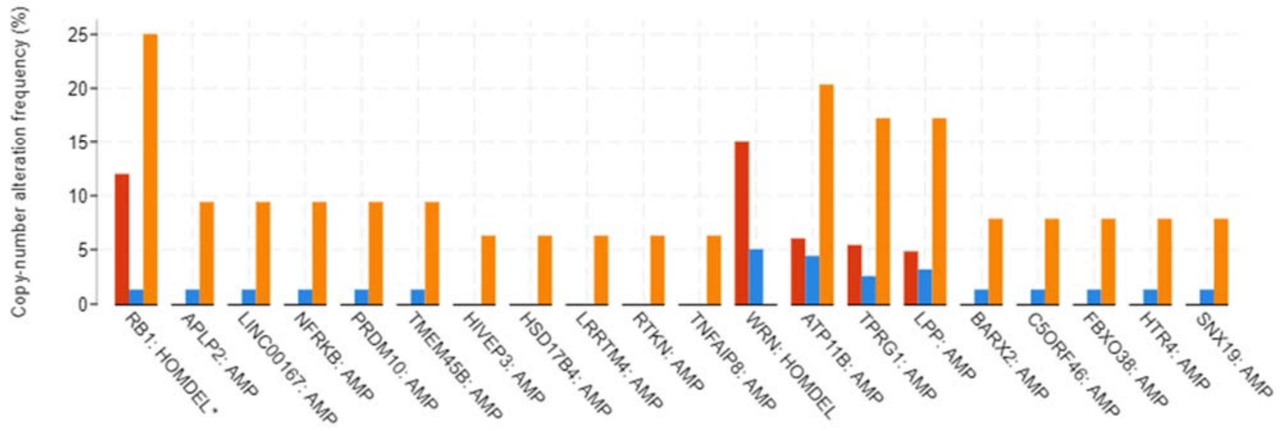

Copy Number in LN, Bone and Liver of 2019 SU2C PNAS Study

Supplemental Figure 5. Distinct copy number alterations in metastatic prostate cancer by metastasis location. The cBIO portal was used to compare the 2019 PNAS metastatic prostate cancer data set by tissue site. All patients were mutually exclusive and not represented in more than one group.

A

Ligands	Antagonists	Receptors/Intracellular mediators
BMP2	NOG	ACVR1
BMP3	GREM1	BMPR1A
BMP4	GREM2	BMPR1B
BMP5	CHRD	BMPR2
BMP6	CHRDL1	TGFBR3
BMP7	CHRDL2	SMAD1
BMP8B	DAND5	SMAD4
GDF2	SOST	SMAD5
BMP10	SOSTDC1	SMAD9
GDF11	NBL1	SMAD6
GDF7	TWSG1	SMAD7
GDF6	BMPER	ID1
GDF5	FST	ID3
BMP15	FSTL1	GATA3

B

Gene	Forward	Reverse
ID1	CTGCTCTACGACATGAACGG	GAAGGTCCCTGATGTAGTCGAT
SMAD6	CCTCCCTACTCTCGGCTGTC	GGTAGCCTCCGTTTCAGTGTA
SMAD7	TTCCTCCGCTGAAACAGGG	CCTCCCAGTATGCCACCAC
BMP3	ACTCCGTGAGACTGAGCCAA	CCTGTCATAGAGCCACAGCATA
MMP8	TTTTGATGCCGAAGAAACATGGA	GTGAGCGAGCCCCAAAGAA
GAPDH	CTGGGCTACACTGAGCACC	AAGTGGTCGTTGAGGGCAATG

Supplemental Figure 6. BMP Pathway genes. A) The BMP pathway can be simplified into ligands, soluble antagonists and intracellular signaling components. B) SYBR qPCR primers used at 60 degrees to validate QIAGEN Osteogenesis qPCR arrays.
